# Historical distribution maps of vascular plants in Northern Europe

**DOI:** 10.1002/ecy.70494

**Published:** 2026-09-22

**Authors:** Matilda Arnell, Alistair G. Auffret, Kristoffer Hylander

**Affiliations:** ^1^ Department of Ecology, Environment and Plant Sciences Stockholm University Stockholm Sweden; ^2^ Bolin Centre for Climate Research Stockholm University Stockholm Sweden; ^3^ Department of Ecology Swedish University of Agricultural Sciences Uppsala Sweden

**Keywords:** distribution maps, historical distributions, range maps, twentieth century, vascular plants

## Abstract

Historical distribution maps are important as baseline data when assessing biodiversity change. Here, we provide a database with digitized distribution maps originally published in *Atlas of the distribution of vascular plants in northwestern Europe* (1971) by Professor Eric Hultén. The database contains georeferenced and digitized distribution maps for 1909 vascular plant species in 616 genera and 119 families. In his work, Eric Hultén compiled spatially explicit data from a vast body of knowledge that had been acquired by professional and amateur botanists. Data were collated from scientific publications, local and regional floras and plant lists, species surveys, and herbarium records. The distribution maps cover the Scandinavian peninsula, including the whole extent of Sweden, Norway, Finland, and Denmark. The Baltic countries Estonia, Latvia, and Lithuania are covered in part, as well as parts of Russia and a small part of northern Germany. Given the scale of the original maps, the effective resolution of the range data is 16 × 16 km. The data on which the original ranges were based varied within the area covered, with comparatively less data available for Russia and the Baltic countries. In order to make this important information compatible with modern analyses, we developed an automated method combined with manual data cleaning to georeference and extract distribution data from the printed maps. The digitized distribution maps are available as spatial polygon features (GeoJSON), which can be converted into gridded or regional occupancy data for each species using the code provided. The digitized polygon range maps in the dataset can be used to illustrate historical distributions of specific species, and when converted to occupancy data, they can be used as baseline distributions in any type of niche or distribution model. In combination with current occurrence records, the historical data can also be used to assess distribution change for the entire assemblage of vascular plants, generating important insights on the drivers of biodiversity change. If, in addition to its scientific and practical value, it can also contribute to keeping the study of »scientia amabilis»—the lovable science, as Eric Hultén himself phrased it—alive, then its purpose has been achieved. The data and code are released under a Creative Commons Attribution 4.0 International (CC BY 4.0) license.

## CONFLICT OF INTEREST STATEMENT

The authors declare no conflicts of interest.

## Supporting information


Data S1.

## Data Availability

Data and code are available as Supporting Information. Additionally, these materials are archived by the Swedish National Data Service with data available at https://doi.org/10.58141/y1x5-cx68 and code available at https://doi.org/10.58141/kvrm-hq58.

