# Supplementary material for: Historical distribution maps of vascular plants in Northern Europe
Source: Ecology. 2026 Sep 22;107(9):e70494. doi: 10.1002/ecy.70494 (PMC13597165; doi:10.1002/ecy.70494)
Supplement: Supplementary file 1 [file ECY-107-e70494-s001.zip › Metadata_S1.pdf]

Data Paper published in *Ecology*, an Ecological Society of America journal

Metadata S1

Historical distribution maps of vascular plants in Northern Europe

Matilda Arnell, Alistair G. Auffret, and Kristoffer Hylander

Class I. Data Set Descriptors

A. Data set identity

Data set of digitized distribution maps of vascular plants in northwestern Europe.

B. Data set identification code

Data: DataS1.zip. The compressed folder contains the following files:
HultenRangeMapsDatabase.zip – digitized distribution maps (.geojson)
HultenMetadata.csv – metadata file
align_bUnwarpJ.ijm – Fiji ImageJ script to align scanned maps
HultenRangeMaps.zip – R package

C. Data set description

1. Originators:

Matilda Arnell
matilda.arnell@su.se

Kristoffer Hylander
kristoffer.hylander@su.se

Department of Ecology, Environment and Plant Sciences
Stockholm university
SE-106 91 Stockholm
Sweden

Alistair G. Auffret
alistair.auffret@slu.se

Department of Ecology
Swedish University of Agricultural Sciences
Box 7070
750 07 Uppsala
Sweden

2. Abstract:

Historical distribution maps are important as baseline data when assessing biodiversity change. Here we provide a database with digitized distribution maps originally published in *Atlas of the distribution of vascular plants in northwestern Europe* (1971) by professor Eric Hultén. The database contains georeferenced and digitized distribution maps for 1909 vascular plant species in 616 genera and 119 families. In his work, Eric Hultén compiled spatially-explicit data from a vast body of knowledge that had been acquired by professional and amateur botanists. Data were collated from scientific publications, local and regional floras and plant lists, species surveys, and herbarium records. The distribution maps cover the Scandinavian peninsula, including the whole extent of Sweden, Norway, Finland and Denmark. The Baltic countries Estonia, Latvia and Lithuania are covered in part, as well as parts of Russia and a small part of northern Germany. Given the scale of the original maps, the effective resolution of the range data is 16×16 km. The data on which the original ranges were based varied within the area covered, with comparatively less data available for Russia and the Baltic countries. In order to make this important information compatible with modern analyses, we developed an automated method combined with manual data cleaning to georeference and extract distribution data from the printed maps. The digitized distribution maps are available as spatial polygon features (GeoJSON), which can be converted into gridded or regional occupancy data for each species using the code provided. The digitized polygon range maps in the dataset can be used to illustrate historical distributions of specific species, and when converted to occupancy data they can be used as baseline distributions in any type of niche or distribution model. In combination with current occurrence records, the historical data can also be used to assess distribution change for the entire assemblage of vascular plants, generating important insights on the drivers of biodiversity change. If, in addition to its scientific and practical value, it can also contribute to keeping the study of »scientia amabilis» - the lovable science, as Eric Hultén himself phrased it - alive, then its purpose has been achieved. The data and code are released under a Creative Commons Attribution 4.0 International (CC BY 4.0) license.

D. Key words/phrases:

Vascular plants, distribution maps, range maps, historical distributions, twentieth century

Class II. Research origin descriptors

A. Overall project description

Here we describe the original data published in the *Atlas of the distribution of vascular plants in northwestern Europe* (Hultén 1950, 1971). In Class V (Supplemental descriptors) we describe the current project where we digitize the distribution maps in the 2nd edition of the atlas (Hultén 1971) producing a database containing historical distribution maps for 1909 vascular plant species in 616 genera and 119 families.

1. Identity: *Atlas of the distribution of vascular plants in northwestern Europe*

2.Originators

Professor Eric Hultén (born 1894, deceased 1981)
Head of Botany (1945-1961), Swedish Museum of Natural History, Stockholm, Sweden

3. Period of study: 1935-1971

4. Objectives
In 1950, Eric Hultén published the first edition of his seminal work on the geographical distributions of vascular plants in northwestern Europe (Hultén 1950). The second edition of the atlas (Hultén 1971) contains distribution maps for 1909 species as well as phenological maps of time of flowering, fruit maturation, leaf-out and senescence for a limited number of taxa. The maps are based on an extensive data collection including obtaining spatial information from regional floras and herbarium records, as well as expert opinions. The following information on the method used by Hultén has been retrieved from the written introduction of the two editions of the atlas, translated from Swedish (Hultén 1950, 1971). The introduction is written in a somewhat anecdotical style which we have tried our best to summarize. The atlas contains a short introduction in English, as well as an English glossary and a translation of the map legend.

Geographic coverage
The distribution maps cover the Scandinavian peninsula ranging approximately 1800 by 1800 kilometres (Figure 1) at a scale of 1:20 000 000. The whole extent of Sweden, Norway, Finland and Denmark are covered. The Baltic countries Estonia, Latvia and Lithuania are covered in part, as well as parts of Russia and a small part of northern Germany.

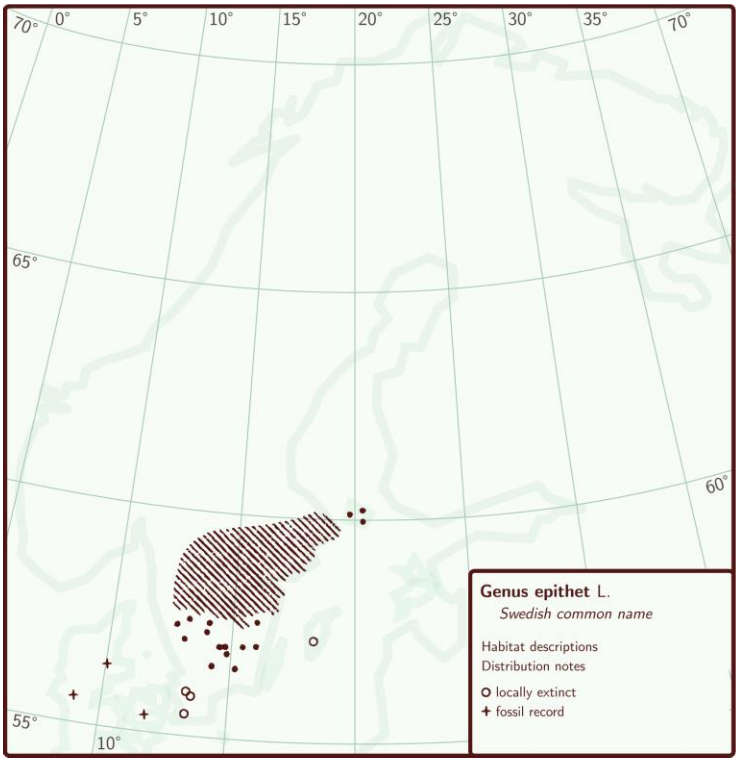

Figure 1. An illustration of the spatial extent and layout of the original range maps in the 2nd edition of the *Atlas of the distribution of vascular plants in northwestern Europe* (Hultén, 1971) using a made-up example range (hatched areas: continuous range, filled circles: isolated finds). The legend in the lower right corner of the original maps contains the species' scientific name and the Swedish common name as well as habitat information and sometimes notes on the distribution range. The legend also explains the meaning of any additional symbols (empty circles and crosses) used in the maps (see Class V - Supplemental descriptors). Image credit: Matilda Arnell (CC BY 4.0).

Occurrence data

The level of available botanical information varied greatly across the mapped area, with the least explored parts found in Russia and the Baltic countries (Table 1). Despite this lack of information, Hultén chose to not leave these parts blank. He believed that there existed enough information to give an overview of the species' distribution in these areas. Areas where the information is based on comparably fewer records are marked on the individual maps (please refer to the *Map legend* section below).

When compiling the available occurrence data, Hultén put together a reference library with all known publications containing notes on where a species had been found. This work commenced 35 years before the publication of the 2nd edition of the atlas. The reference library which was originally placed at the Swedish Museum of Natural History in Stockholm has unfortunately not been recovered. For a description of the reference library and herbarium records used per country, please refer to Table 2. The library contained local and regional floras, species lists covering different areas as well as publications concerning individual species. Based on this literature as well as herbarium records, Hultén derived occurrence records as well as estimations of how common species were in different parts of their ranges (please refer to the *Map legend* section below for further information). In addition to the reference library, an archive of already published distribution maps was compiled. The archive that contained thousands of distribution maps, also established at the Swedish Museum of Natural History, has not been recovered. In total, the compiled available occurrence records probably contained records from the 18th century up until the publication year of the second edition of the atlas in 1971.

Table 1. The level of available botanical information by country listed in the introduction to the *Atlas of the distribution of vascular plants in northwestern Europe* (Hultén 1971).

Country	Well-studied regions	Less well-studied areas
Sweden	Blekinge, Öland, Bohuslän, Stockholm area, Uppland, Dalarna	Småland, Dalsland, northern Värmland, the inner parts of Södermanland, the borders between Dalarna, Gästrikland and Hälsingland, the northern coniferous region between the coast and the mountain range
Norway	Oslo area, the south coast and north coast (swe: sydkusten samt de nordliga delarna)	Østfold, the inner parts of Telemark and Aust-Adger, the border to northern Jämtland (Sweden)
Finland	Åland, south coast, Karelia, the Bothnian coast	the inner parts of the country except the southern lake district, some regions in the northern parts of the country
Denmark	The whole country	
Baltic countries	All areas less well studied compared to the Scandinavian countries	The inner parts of Courland
Russia	Karelia, Kola peninsula, Khibiny Mountains (Swe: Umptekområdet). All areas less well studied compared to the Scandinavian countries. *General difficulties in obtaining information*	Most parts little explored. *General difficulties in obtaining information.*

However, Hultén's aim was to accurately map the species current (1970) distribution, and old, unreliable records would probably not have been included. On some maps, old and unreliable records have been noted with specific symbols.

Turning records of occurrences into distribution maps

The painstaking process of mapping the compiled tens of thousands of occurrence records onto maps started twelve years before the first publication of the atlas. At this time, the spatial information available would typically have been a name of a village or parish or the name of a specific land feature, such as a mountain. Each such occurrence record was located and marked with a dot on draft maps (Swe: *arbetskartor*) with the scale of 1:15 000 000. On these draft maps, the different sources of data (regional floras, herbarium records etc.) were marked in different colours to aid the final validation when turning the draft maps into manuscript maps (Swe: *manuskiptkartor*) at the final scale of 1:20 000 000. The draft maps were then photocopied and sent to a number of botanists and biogeographers around Scandinavia, Germany and the Baltic countries who contributed with expert knowledge (a complete list of the experts is available in the end of the original introduction). The data on the draft maps were manually transferred to the

manuscript maps using light tables. The validation process and production of manuscript maps took two years. Finally, the data on the manuscript maps were transferred onto maps suitable for printing. Despite substantial effort devoted to locating the draft maps and the manuscript maps, these have unfortunately not been recovered.

Map resolution and legend

The original size of each printed distribution map is 89×91 mm. At the scale of 1:20 000 000 the dots on the printed maps marking isolated finds are approximately 0.8 mm in diameter (Figure 1) which is equivalent to 16 km on the ground. The effective resolution of the maps is thus 16×16 km. Please refer to Table 3 for exceptions. Hultén states three reasons for choosing such a low resolution: economy, to render the book easy to handle and to clearly mark the character of the maps as overview maps.

Table 2. Description of the Hultén reference library and herbarium records by country listed in the introduction to the 1st edition of the *Atlas of the distribution of vascular plants in northwestern Europe* (Hultén 1950).

Country	Main references	Herbarium records
Sweden	Comprehensive reference library. References listed in *Bibliotheca botanica suecana* (Krok 1925) was used as a starting point.	The herbarium at the Swedish Museum of Natural History in Stockholm, the herbarium at the Lund Botanical Museum, the Scandinavian herbarium in Lund.
Norway	Comprehensive reference library.	The herbarium at the Natural History Museum in Oslo.
Finland	Main reference: *Conspectus Florae Fennicæ* (Hjelt 1888). Complemented with a reference library of the Finnish botanical literature published after 1888.	The herbarium at the Museum of Natural History in Helsinki.
Denmark	Main references: *Karplanternes Udbredelse i Danmark* (Jessen 1926) and *Den danske Flora* (Rostrup and Jörgensen 1947).	
Baltic countries	Reference library. Scarce botanical literature compared to the Scandinavian countries, wherefore also literature on for example nature conservation was included.	
Germany	Main references: Flora von Ostpreussen (Steffen 1940) and expert knowledge provided by dr. Willy Christiansen, Kiel.	
Russia	No listed references. The *Flora of the USSR* (Komarov 1934) may have been used.	

The maps contain information on isolated observations, referred to as isolated finds by Hultén, (represented by dots on the maps) as well as continuous distributions (represented by hatched areas) (Table 4 and Figure 2). As for the isolated observations, the delineation of the continuous distributions was based on the compiled occurrence data (see the section Occurrence data above).

The hatched areas visualise areas where the locations of the isolated finds generally occurred closer together than 16 km. In areas where the species was known to be common the hatched lines are drawn in bold (Table 4 and Figure 2). In areas where fewer data than average was available (see Table 1) continuous distributions were drawn with broken lines (Swe: *avbruten sträckning*. Eng: dotted lines).

Hultén acknowledges that, since not all localities were known, the decision of where exactly to draw a continuous distribution as opposed to isolated finds was based on his, and his colleagues, expert opinions informed by the compiled occurrence records. (Swe: *Eftersom alla lokaler ej är kända, blir de gränser, där sträckningarna börjar, en omdömessak, som jag med ledning av det på arbetskartorna sammanställda materialet fått utforma för varje särskilt fall.*)

In addition to the dots marking isolated finds, some maps contain two additional symbols: empty circles and crosses (+; see Figure 1). These symbols mark different types of occurrence records in different maps. Their meaning is explained in the legend in the lower right corner of each map. Empty circles typically indicate localities where a species has gone locally extinct (Swe: *utgången*). Crosses typically mark fossil findings.

Table 3. The resolution of the distribution data by country in the 2nd edition of the *Atlas of the distribution of vascular plants in northwestern Europe* (Hultén 1971).

Country	Resolution
Sweden	Approximately 16 × 16 km.
Norway	Approximately 16 × 16 km.
Finland	Approximately 16 × 16 km.
Denmark	The 52 districts of the Danish topographical-botanical survey (in Danish: Topografisk-Botanisk Undersøgelse af Danmark). The size of the Danish districts ranges from approx. 112 km^2 to 5568 km^2 with a mean size of 2400 km^2, which could roughly be considered as a resolution of 47 × 47 km.
Baltic countries	Approximately 16 × 16 km, although based on less information compared to the Scandinavian countries (see Table 1).
Germany	Approximately 16 × 16 km, although based on less information compared to the Scandinavian countries (see Table 1).

Some maps are marked with question marks (Figure 3). Hultén gives no explanation for this symbol. They may represent uncertain localities that were not changed or removed from the manuscript maps during the validation process and were thus printed accidentally. However, since Hultén spent two years validating the maps one may also assume that he wanted to point out localities with uncertainties to the reader.

Table 4. Symbols and explanations from the legend in the *Atlas of the distribution of vascular plants in northwestern Europe* (Hultén, 1971). Image credit: Matilda Arnell (CC BY 4.0).

Symbols	Explanation
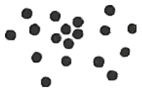	**Isolated finds**<br>The dots are about 0.8 mm in diameter on the maps, each corresponding to 16 kilometres on the ground. Consequently, if the finds are closer to one another than 16 kilometres, the dots are confluent on the maps. Only if this is the case within small areas have the occurrences been marked with confluent dots. Similar areas of larger size have been hatched instead.
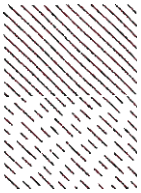	**Less common - fairly common**<br>If the finds are considered on an average to lie closer to one another than 16 km, although the species cannot be denoted as common within the area it has been hatched with tine lines. Dotted lines indicate that fewer data than usual have served as a basis for estimating the degree of frequency.
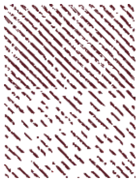	**Common**<br>If the species is considered to be common or very common within the area, the latter has been hatched with thick lines. In this case also, dotted lines have been used to indicate that fewer data than usual have served as a basis for estimating the degree of frequency.

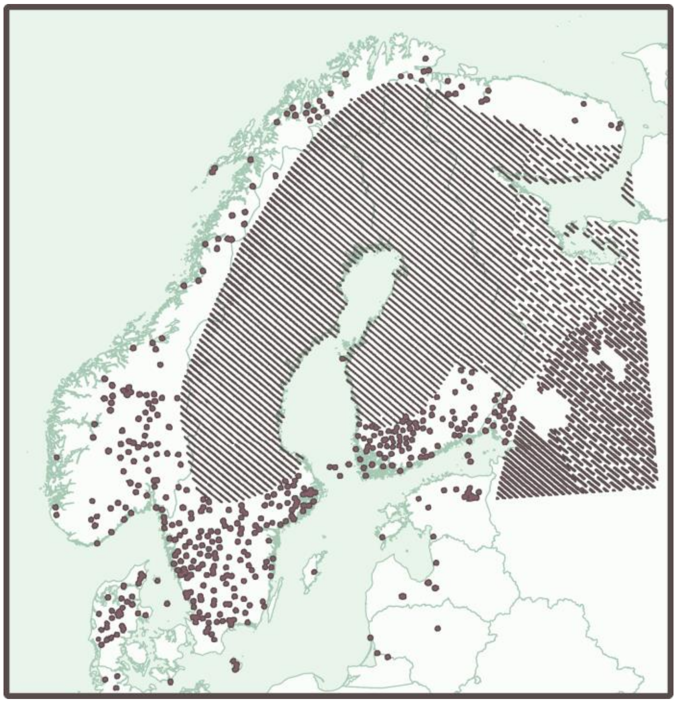

Figure 2. An example of a map with all five types of distribution symbols (Table 4). Dots are used to mark isolated finds. Hatched lines mark the parts of the range where the species is less common to fairly common (parts of Sweden, Finland and northwestern Russia). Bold hatched lines mark the part of the range where the species is common (parts of western Russia). Broken hatched lines mark parts of the range where fewer data than average was available (parts of Russia in the eastern part of the mapped distribution). The distribution map is recreated using the digitized range (see Class V - Supplemental descriptors) of *Lycopodium complanatum* from the 2nd edition of the *Atlas of the distribution of vascular plants in northwestern Europe* (Hultén 1971). Image credit: Matilda Arnell (CC BY 4.0).

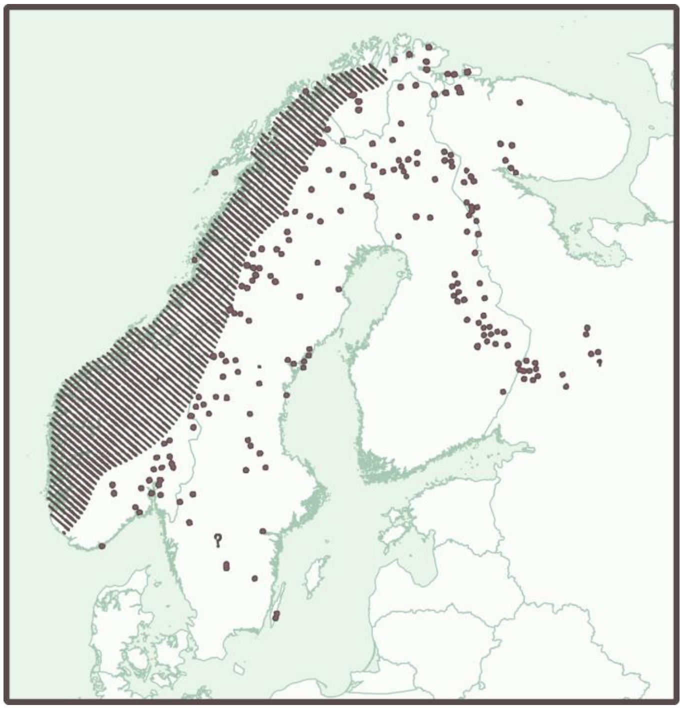

Figure 3. An example of the use of question mark symbols, located in southern Sweden. Hultén gives no explanation for this symbol. The distribution map is recreated using the digitized range (see Class V - Supplemental descriptors) of *Asplenium viride* from the 2nd edition of the *Atlas of the distribution of vascular plants in northwestern Europe* (Hultén, 1971). Image credit: Matilda Arnell (CC BY 4.0).

Phenological data
As well as geographic distributions, Hultén's work also contains maps showing spatial patterns in phenological events (flowering time, leaf out time, timing of leaf abscission and timing of fruit maturation) for 47 species (Table 5). The phenological data for Sweden was obtained from the works by Hans Wilhelm Arnell, Knut Arnell, and Sigfrid Arnell (Arnell 1923, 1927, Arnell and Arnell 1930). For Finland, the data was obtained from the works by Adolf Moberg (Moberg 1860, 1885) as well as later phenological observations published in the book series *Bidrag till kännedom av Finlands natur och folk* published by the The Finnish Society of Sciences and Letters. For areas where there was a lack of phenological observations, Hultén complemented the data from published sources with phenological data from herbarium specimens. Given the variability of the data, the isobars on the phenological maps generally have an interval of 14 days (Figure 4).

While Hultén acknowledges the difficulties in turning such temporally varying phenomenon as flowering time and senescence into maps, he believed that the maps could nonetheless be of interest to the general public. Since the phenology maps are not range maps per se, they have not been included in this database.

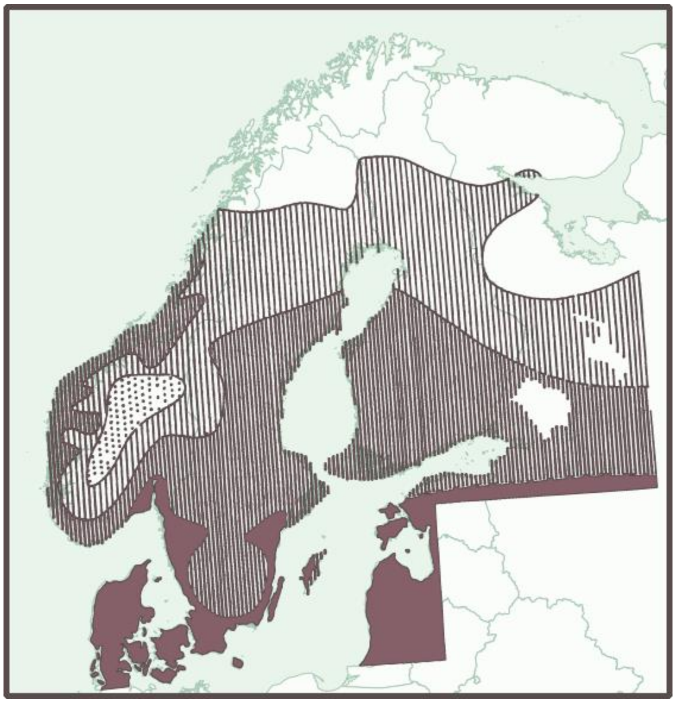

Figure 4. Example of a phenological distribution map. The map is recreated using the digitized spatial patterns in phenological events (see Class V - Supplemental descriptors) of the flowering time of *Convallaria majalis* from the 2nd edition of the *Atlas of the distribution of vascular plants in northwestern Europe* (Hultén 1971). The different areas show how the onset of flowering differs across Scandinavia. The flowering time is approximately three weeks. Solid color: last week of May. Densely hatched: first half of June. Sparsely hatched: Latter half of June. Dotted area: July. The rectangular area without phenological data is where the legend was located in the original map. Image credit: Matilda Arnell (CC BY 4.0).

Table 5. Type of phenological maps and list of species per phenological map category in the 2nd edition of the Atlas of the distribution of vascular plants in northwestern Europe (Hultén 1971).

Type of phenology record	Scientific name
flowering time	*Calla palustris, Convallaria majalis, Cypripedium calceolus Dactylorhiza maculata subsp. Fuchsia, Epipogium aphyllum, Calypso bulbosa, Salix caprea, Corylus avellana, Honckenya peploides, Dianthus superbus, Nuphar lutea, Thalictrum alpinum, Caltha palustris, Trollius europaeus, Hepatica nobilis, Anemone nemorosa, Ficaria verna, Corydalis intermedia, Cochlearia officinalis, Draba verna, Chrysosplenium alternifolium, Filipendula ulmaria, Prunus padus, Rubus chamaemorus, Sorbus aucuparia, Dryas octopetala, Lathyrus japonicus subsp. maritimus, Geranium sylvaticum, Tilia cordata, Vaccinium vitis-idaea subsp. vitis-idaea, Calluna vulgaris, Primula veris, Lysimachia europaea, Lysimachia maritima, Galium verum, Linnaea borealis, Tanacetum vulgare, Tussilago farfara, Centaurea cyanus*
leaf out time	*Betula pubescens, Betula pendula*
timing of leaf abscission	*Betula pubescens, Betula pendula*
timing of fruit maturation	*Rubus idaeus, Fragaria vesca, Vaccinium myrtillus, Vaccinium vitis-idaea subsp. vitis-idaea*

5. Abstract

Below follows the original abstract from *Atlas of the distribution of vascular plants in northwestern Europe* (Hultén 1971) translated to English.

Botanically, the Nordic region is certainly the best-known area in the world of comparable size. The legacy of Linnaeus, nurtured in Swedish schools right up to the present day in the form of mandatory plant collection and learning of Latin plant names, has made many Swedes interested amateur botanists, who, alongside their profession, have found a lifelong interest in botanical studies and excursions. The strong interest in nature, which is considered a typical Nordic trait, and the increasingly intense tourism have worked in the same direction. Even our northernmost mountain regions are now so well researched that the discovery of a new plant there is a great sensation that attracts attention even outside the circle of specialists. A vast body of knowledge has been collected by Nordic botanists and amateur botanists over two hundred years. It has been scattered in several thousand large and small publications, in landscape floras and plant lists, in local floras, species surveys and occasional notices or hidden in herbaria. During the last three decades, a large but not very uniform map material has also been published, mostly of plants that in one way or another have been considered notable. Many times, a single one of these maps has required a review of a considerable part of the botanical literature. Mapping individual, more general species in this way requires an enormous amount of work compared to the result. Even for the specialist botanist, it has often been difficult to form an opinion about the distribution of a species in the Nordic countries without unreasonable effort. Most collectors have more or less collected blindly. They have time and again spent time and work collecting plants in places from which they are well known, while on the other hand they have passed over them in places from which they are unknown both in herbaria and literature. The reason for this is obvious. No work that provides a general compilation of the distribution of Nordic plants has so far been available to the specialist botanist and the general public. This atlas has been created to fill this gap in our knowledge and in our literature. If, in addition to its scientific and practical value, it can also contribute to keeping the study of »scientia amabilis» - the lovable science - alive in the Nordic and Swedish tradition, then its purpose has been achieved.

6. Sources of funding: Grant from Knut and Alice Wallenberg Foundation awarded to Professor Eric Hultén in 1945.

Class III. Data set status and accessibility

A. Status

The database was last updated on 10 April 2026. Metadata (this document) was last modified on 25 August 2026.

B. Accessibility

Data and code are archived in the Supporting Information (DataS1.zip), as well as on the Swedish National Data Service (data: Arnell et al. 2026, https://doi.org/10.58141/y1x5-cx68; code: Arnell 2026, https://doi.org/10.58141/kvrm-hq58). Contact Matilda Arnell (matilda.arnell@su.se) with any questions. Data and code are released under a Creative Commons Attribution 4.0 International (CC BY 4.0) license. When using this data, please cite both the dataset (Arnell et al. 2026), the original publication (Hultén 1971) and this accompanying data paper.

Class IV. Data structural descriptors

A. Data set files

Distribution maps are provided as one polygon spatial feature file (GeoJSON) per species. The file names of the distribution map files are named after the current accepted scientific name of the species and the original atlas map number (e.g. *Lycopodium alpinum_00001.geojson*).

The data set also contains a metadata file containing information on the original species names and atlas map number as well as how the accepted scientific name was matched to the original species name listed in the atlas, and if any non-occurrence records have been manually removed from the map. For details, see Class V, part A. –*Manual data cleaning* and *Updated scientific nomenclature*. The data set also contains a script used to align the scanned maps and an R-package *HultenRangeMaps* with a manual and scripts to perform the automated digitalisation process. To aid spatial analyses, the package provides a function to match the digitized distribution maps to any grid stored as a polygon spatial feature. For details, see Class V, part A. – *Automated image alignment and georeferencing* and part D. – *Computer programs and data-processing algorithms*. The details of the data set files are described in table 6.

Table 6. Details of the data set files.

file	Number of records	file format
HultenRangeMapsDatabase.zip	1909 files	.geojson
HultenMetadata	1909 rows (excluding header) and 16 columns	.csv
align_bUnwarpJ.ijm	1 text file	.ijm
HultenRangeMaps.zip	R package	

B. Variable information

Explanations for the variables (columns) in the metadata file (HultenMetadata.csv) are listed in table 7. For details, see Class V, part A. - *Updated scientific nomenclature* and *Manual data cleaning*.

Table 7. Explanations for the variables in the metadata file HultenMetadata.csv. For details on how the variables *scientificName*, *dyntaxa*, *dyntaxaID*, *nameStatus*, *nameStatusRef*, *refDetails*, *mergedRanges*, *mergedRangesNote* were generated, see Class V, part A. - *Updated scientific nomenclature*. For details on how the variables *manualDataCleaning*, *dataCleaningNote*, *dataCleaningNoteSwe*, *identifiedNotRemoved*, *notRemovedNote*, *notRemovedNoteSwe* were generated, see Class V, part A. - *Manual data cleaning*.

**Variable**	**Explanation**
scientificName	Accepted scientific name. For all species listed in the Swedish Taxonomic Database Dyntaxa (SLU Artdatabanken 2022) the accepted name was retrieved from there. For species not listed in Dyntaxa, we have listed the scientific names accepted by the Global Biodiversity Information Facility (GBIF.org 2022).
speciesHulten	Scientific name when the atlas was printed in 1971 (Hultén 1971).
numberHulten	Map number in the original publication (Hultén 1971).
dyntaxa	Indicates if the species is listed in the Swedish Taxonomic Database Dyntaxa (SLU Artdatabanken 2022).<br><br>*1* - listed in Dyntaxa<br><br>*0* - not listed in Dyntaxa
dyntaxaID	Taxon identification number in the Swedish Taxonomic Database Dyntaxa (SLU Artdatabanken 2022).
nameStatus	Indicates if the scientific name has been constant since 1971.<br><br>*constant* - scientific name has been constant since 1971<br><br>*synonym* - the name in the atlas is a scientific synonym
nameStatusRef	Reference for how the accepted scientific name was matched to the species name listed in the atlas (Hultén 1971).<br><br>*Dyntaxa* - all species listed in the Swedish Taxonomic Database was matched using Dyntaxa (SLU Artdatabanken 2022)

	POWO - scientific names and synonyms with no match in Dyntaxa were checked using the Plants of the world online database (POWO 2022). The accepted names from POWO were then matched again in Dyntaxa<br><br>GBIF - scientific names and synonyms with no match in either Dyntaxa or POWO were checked using the GBIF database (GBIF.org 2022). The accepted name from GBIF was then matched again in Dyntaxa<br><br>*Hulten* - For scientific synonyms with no match in either Dyntaxa, POWO or GBIF, we checked the atlas (Hultén 1971) for listed scientific synonyms as well as synonyms listed in a later atlas published by Hultén (Hultén and Fries 1986), cross-checking these synonyms in POWO and GBIF. The accepted names from POWO were then matched again in Dyntaxa.
refDetails	Short description of how the name has changed and/or the reference for how the match between the scientific synonym and the accepted scientific name was established.
mergedRanges	Some species, subspecies and varieties that were historically considered separate are now considered to belong to the same species. When this was the case, we merged the ranges into one polygon spatial feature file, see the section<br><br>*1* - the range consists of merged ranges of two or more historical species<br><br>*0* - the range has not been merged
mergedRangesNote	Information on the species, subspecies and varieties that were merged.
manualDataCleaning	Some maps were identified to contain symbols (crosses, open circles or lines) with additional information noted in the legend of the map. These records were manually removed if they represented non-occurrences..<br><br>*1* - non-occurrence records have been removed<br><br>0 - no records have been removed
dataCleaningNote	Information on the type of non-occurrences records removed. The categories are based on the additional information (in Swedish) noted in each map legend. When maps contain more than one type

	of non-occurrence record they are listed separated by semicolons (;). The column *dataCleaningNoteSwe* contains the Swedish word or sentence used to describe the non-occurrence record.<br><br>*fossil record* - record described as fossil record<br><br>*locally extinct* - record described as locally extinct<br><br>*range outline* - line outlining ranges of varieties, subspecies, hybrids or uncertain range limits. The column *dataCleaningNoteSwe* contains information on the on the specific varieties, subspecies, hybrids etc. when noted.<br><br>*unreliable record* - record described as unreliable<br><br>*question mark* – question marks with no information in the map legend
dataCleaningNoteSwe	The categories in the *dataCleaningNote* column are based on the additional information noted in the map legend in Swedish. Here we list the Swedish word or sentence used to describe the non-occurrence record on which the categories are based.
identifiedNotRemoved	Some maps were identified to contain symbols (crosses, open circles or lines) with additional information noted in the legend of the map. These records were kept if they represented specific types of occurrence records.<br><br>*1* – contains records with additional information that has not been removed<br><br>*0* – no records with additional information identified
notRemovedNote	Information on the type of occurrence record that was identified but not removed. The categories are based on the additional information (in Swedish) noted in each map legend. When maps contain more than one type of specific occurrence record they are listed separated by semicolons (;). The column *notRemovedNote Swe* contains the Swedish word or sentence used to describe the specific type occurrence record.<br><br>*introduced* – records described as introduced<br><br>*transient* – records described as transient

	naturalized – records described as naturalized<br><br>*hybrid* – records of hybrids. The column *notRemovedNoteSwe* contains information on the specific variety when noted.<br><br>*variety* - records of varieties. The column *notRemovedNoteSwe* contains information on the specific variety when noted.<br><br>*subspecies* - records of subspecies. The column *notRemovedNoteSwe* contains information on the on the specific subspecies when noted.<br><br>*no information* - symbols with no information in the map legend
notRemovedNoteSwe	The categories in the *notRemovedNote* column are based on the additional information noted in the map legend in Swedish. Here we list the Swedish word or sentence used to describe the specific occurrence record on which the categories are based.

Class V. Supplemental descriptors

A. Data acquisition

1. Data forms or acquisition methods

In order to make the information in the printed maps compatible with modern analysis methods we performed three main steps: scanning and clipping, aligning and georeferencing and extraction of distribution data. Given the large number of maps, we developed an automated method for aligning, georeferencing and extracting distribution data from the scanned maps. We also performed some manual data cleaning. Additionally, we matched the scientific names in the atlas to the Swedish taxonomic database Dyntaxa (SLU Artdatabanken 2022) in order to attain an updated scientific nomenclature. Some species, subspecies and varieties that were historically considered separate are now considered to belong to the same species. When this was the case, we merged their ranges into one range. The process is described in detail below.

Automated image alignment and georeferencing

Georeferencing is the process of assigning coordinates of a known coordinate reference system to each pixel of the scanned map. This is done by choosing points on the map corresponding to ground control points (GCPs). Ground control points are points on the surface of the earth with known coordinates. In this project we choose 16 GCPs at the longitudes and latitudes marked on the scanned maps (Figure 5). These 16 points were complemented with 4 GCPs in areas where locations on the printed map deviated from the actual locations (the coast of Norway and Denmark

and the island of Gotland). The manual georeferencing was performed in QGIS (QGIS Development Team 2025).

To automatize the georeferencing process, the manually chosen GCPs are simply assigned to each map. However, high accuracy in the automated georeferencing can only be attained if the maps are perfectly aligned. Due to the manual printing process in 1970, this was not always the case (Figure 6). Given the small scale of the maps a 1 mm offset of a map to the reference map results in an approximate 20 km offset in the georeferenced maps. This problem was reduced by aligning the images using the plugin program bUnwarpJ (Sorzano et al. 2005, Arganda-Carreras et al. 2006) in the image processing program Fiji ImageJ (Schindelin et al. 2012). In this registration process, we aligned each map image (source image) to the reference map (reference image) used when manually assigning the GCPs. The script used in Fiji ImageJ (*align_bUnwarpJ.ijm*) is included in the Supporting Information (DataS1.zip).

After aligning the images, the maps were georeferenced using gdal functions (GDAL/OGR contributors 2025) via the package gdalUtilities (O'Brien 2023) in R (R Core Team 2025).

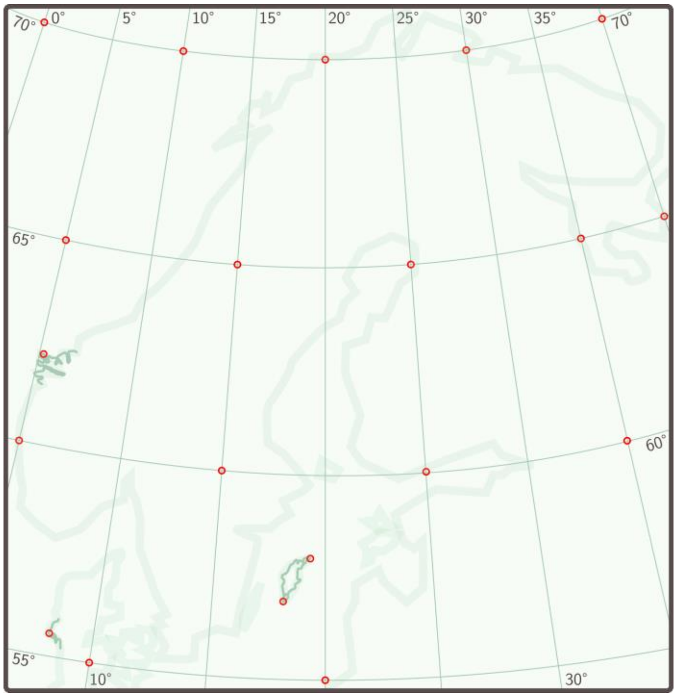

Figure 5. Twenty manually assigned ground control points (GCPs) used in the automated image alignment. Empty circles: ground control points. Image credit: Matilda Arnell (CC BY 4.0).

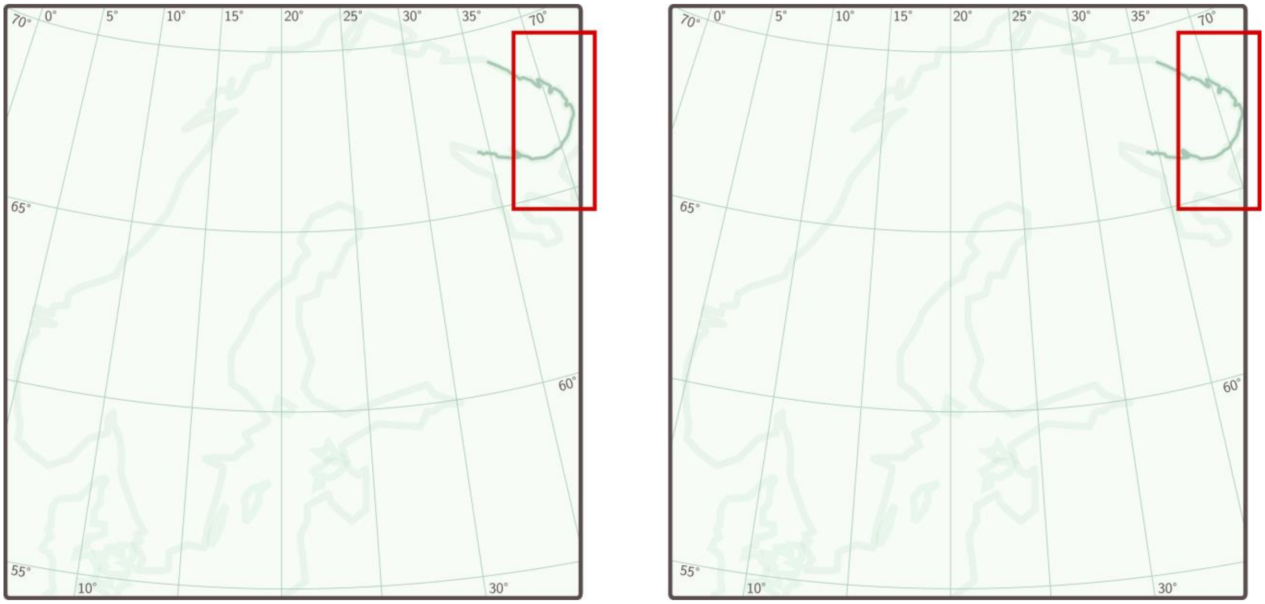

Figure 6. A one-millimeter offset, highlighted by the red square in the upper right corner of each map, will result in an approximate 20 km offset in the georeferenced maps. This is an illustration of a potential offset between two maps. For an example of an offset like the one that is exemplified here, compare the distribution maps of *Saussurea amara* and *Equisetum fluviatile* in the 2nd edition of the *Atlas of the distribution of vascular plants in northwestern Europe* (Hultén 1971). Image credit: Matilda Arnell (CC BY 4.0).

Automated extraction of distribution data

The maps were printed in only two colours with presence of a species represented in red and geographical borders represented in green. We could therefore extract the distribution data from the georeferenced raster images by extracting the red pixel values. The digitalization process was performed in R (R Core Team 2025).

Automated data cleaning and conversion from raster to spatial features

After extracting the distribution data, we removed the border, map number and legend (also represented in red on the maps) in an automated procedure where a polygon was used to crop and mask out non-range parts of the extracted data. Given that this was an automated procedure, with the same polygon used for all maps, the precision was not perfect for all extracted ranges. For ranges that extended all the way to the eastern or south-eastern part of the mapped area (where the map border or legend meets the land and not water) small parts of the range areas were also removed. This was an unintentional result of weighing precision against the time it would have taken to remove the map border, map number and legend manually for each map.

To remove small digitalization errors we identified ‘clumps’ of connected cells and removed clumps with <15 cells using the clump() function in the *raster* package (Hijmans 2025a). We then converted rasters to polygon features using the as.polygons() function in *terra* (Hijmans 2025b).

The polygon features were saved as one GeoJSON file per species using the geojson_write() function in the *geojsonio* (Chamberlain et al. 2025), with coordinates given in the coordinate reference system WGS 84. GeoJSON is an open standard format for representing geographical features and WGS 84 (World Geodetic System, version 84) is a unified geodetic system for the whole world.

The files in the database are thus typical range maps with polygons representing specie's presence (Figures 2, 3 and 8). These polygons are very useful for visualizations and manual visual interpretations of ranges, since they preserve all the information from the original maps, explained in the original map legend (Table 4). As an example, the digitized range of *L. complanatum* (Figure 3) visualises all five types of distribution symbols. On the original map as well as in the digitized polygon features, dots mark isolated occurrences, meaning that individuals or populations are generally 16 km apart or more (Denmark, the Baltic countries, southern Sweden and Finland and most of Norway). Hatched lines mark the parts of the range where the species is less common to fairly common (northern Sweden and Finland and northwestern Russia). Bold hatched lines mark the part of the range where the species is common (parts of western Russia). Broken hatched lines – both thin and bold – mark parts of the range where fewer data than average was available (parts of Russia in the eastern part of the mapped distribution).

To aid spatial analyses, we also provide an R function to match the digitized distribution maps to any grid or set of regions stored as a polygon spatial features (described in *D. Computer programs and data-processing algorithms* below).

It is important to note that the nature of the original data, for example dots marking isolated occurrences on small islands, sometimes results in ranges that extend past land borders into water for terrestrial plants. Depending on the application of the data, this may need to be corrected for prior to analyses.

Manual data cleaning

Some maps were identified to contain symbols (crosses, open circles or lines) with additional information noted in the legend of the map (see Figure 1 and 8). These records were manually removed if they represented non-occurrences (fossil records, locally extinct individuals or populations, unreliable records or lines outlining the ranges of subspecies or varieties) and kept if they represented specific types of occurrence records (e.g. naturalized, introduced or transient individuals or populations). Some maps contained question marks with no explanation in the legend or anywhere else in the text. All question marks were removed. Some maps contained open circles with no information in the map legend. These records were kept since open circles usually represented specific types of occurrences, such as naturalized individuals or populations.

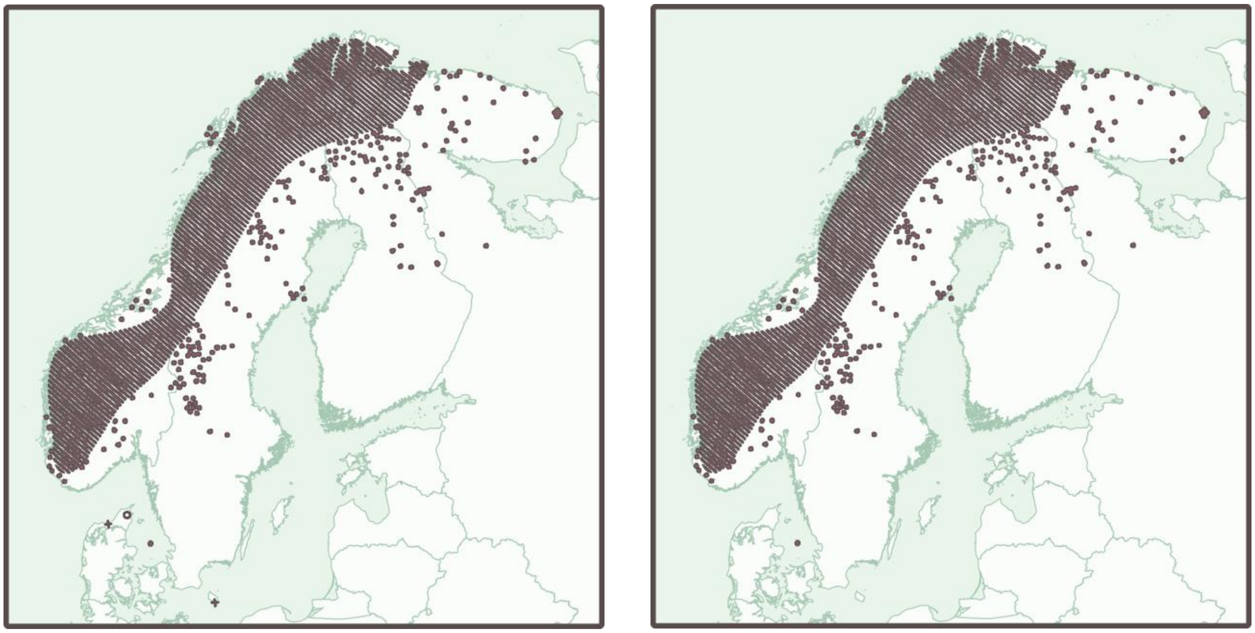

Figure 7. Digitized distribution (dark red polygons representing species presence) after automated data cleaning (left panel) and manual data cleaning (right panel), The distribution maps are recreated using the digitized range (see Class V - Supplemental descriptors) of *Lycopodium alpinum* from the 2nd edition of the *Atlas of the distribution of vascular plants in northwestern Europe* (Hultén 1971). Providing the digitized range maps as polygons in the database means that all the original range information is preserved. Note that the non-occurrence records, one open circle representing a locally extinct individual or population on mainland Denmark and two crosses representing fossil records, one on mainland Denmark and one on the Danish island Bornholm, have been manually removed from the final database map (left panel). Image credit: Matilda Arnell (CC BY 4.0).

Maps with records removed are flagged in the column *namualDataCleaning* (1/0) in the meta data file HultenMetadata.csv (Supporting Information: DataS1.zip). The column *dataCleaningNote* describes the type of record removed (*fossil record*; *locally extinct*; *range outline*; *unreliable record; question mark*). The column *dataCleaningNoteSwe* lists the various Swedish expressions listed in the map legends that we have summarized into the categories of records removed (table 8). Maps with records that were identified as representing specific types of occurrences (crosses or open circles) but not removed are flagged in the column *identifiedNotRemoved* (1/0). The column *notRemovedNote* describes the type of record identified but not removed (introduced; transient; naturalized; hybrid; variety; subspecies; no information). The column notRemovedNoteSwe lists the various Swedish expressions listed in the map legends that we have summarized into categories of records kept (table 9).

Manual data cleaning was performed in QGIS (QGIS Development Team 2025). Small parts of the map border that were not removed in the automated data cleaning process were also removed manually

Table 8. List of the type of records that were identified and removed based on the information in each map legend. The column *dataCleaningNoteSwe* in the metadata file HultenMetadata.csv (Supporting Information: DataS1.zip) lists the various expressions used by Hultén (1971) that we have summarized into the categories listed in the column *dataCleaningNote*.

dataCleaningNote	dataCleaningNoteSwe
fossil record	”*fossil*”, ”*pollenfossil*”, ”*fossil, senkvartär*”, ”*fossilfynd utanför nuvarande utbredningsområdet*”, ”*fossil, sista interglacialtiden*”, ”*fossil, huvudsakligen från Ancylus-tid men även från Litorina-tid*”, ”*fossil utanför nuvarande gräns*”, ”*fossil, senglacial, sporer*”, ”*fossil, senglacial*”, ”*senglaciala fossilfynd, interglaciala fossilfynd*”, ”*pollenfossil från senglacial tid*”, ” *fossil senkvartär (pollen)*”, ”*fossilt pollen, makrofossil*”, ”*pollenfossil från postglacial värmetid utanför nuvarande gräns*”, ”*senglaciala pollenfynd*”, “*postglaciala fossilfynd, interglaciala fossilfynd*”, ”*pollenfynd från senglacial tid av Onobrychis-art*”,”*fossil i Dryas-lera*”, ”*uppgives som fossil*”, ”*pollenfynd från postglacial värmetid*”, ”*subfossil*”, ”*subfossil sporer*”
locally extinct	”*utgången*”, ”*vildväxande till på 1700-talet*”, ”*ej sedd i senare tid*”, ”*ej återfunnen*”, ”*tillfälligt införd, nu utgången*”, ”*förgäves eftersökt*”, ”*ej sedd sedan …*”, ”*troligen utgången*”, ”*osäker eller utgången*”, ”*utdöd*”, ” *utgången nyligen*”, ”*utrotad*”,” *tillfälligt inkommen, utgången*”, ” *ej återfunnen eller utgången*”, ”*tillfällig, numera utgången”, ”osäker uppgift från 1819, ej återfunnen”, ”troligen ej inhemsk, numera utgången*”, ”*osäker eller utgången*”, ”*numera utdöd*”, ”*förr, nu utgången*”, ”*avfallsplatser, nu utgången*”, ”*utgången eller troligen utgången*”, ”*införd, numera utgången*”, ”*äldre uppgifter, (numera utrotad)*”, ” *äldre uppgifter, ej återfunnen*”, ”*utrotad i slutet av 1930-talet*”, ”*ett träd, dött på 1820-talet*”, ”*enligt uppgift funnen på 1870-talet, nu försvunnen*”, ”*nyligen utgången*”, ”*veterligen utgången*”, ”*troligen utgången, senast sedd …*”, ”*utgången sedan mitten av 1800-talet*”, ”*uppgiven men ej återfunnen*”, ”*äldre uppgift, ej återfunnen*” ”*adventiv i åker, utgången*”, ”*beläggexemplar saknas, troligen utgången*”
unreliable record	”*osäker uppgift*”, ”*gammal, osäker uppgift*”, ”*levande rotstock funnen i stranddrift*”, “*äldre osäker uppgift*”, ”*osäker uppgift*”, ”*osäker eller utgången*”, ”*tvivelaktig fyndort*”, ”*osäker*”, ”*osäkra uppgifter*”, ”*äldre uppgift*”
range outline	Line outlining ranges of varieties, subspecies, hybrids or uncertain range limits. The note in Swedish contains information type of record.
question mark	No information in any map legend or elsewhere in the text.

Table 9. List of the type of records that were identified and not removed, based on the information in each map legend. The column *notRemovedNoteSwe* in the metadata file HultenMetadata.csv (Supporting Information: DataS1.zip) lists the various expressions used by Hultén (1971) that we have summarized into the categories listed in the column *notRemovedNote* column.

**notRemovedNote**	**notRemovedNoteSwe**
naturalized	”*förvildad*”, ”*införd förvildad*”, ”*planterad och förvildad*”, ”*förvildad eller på barlast*”, ”*subspontan*”, ”*odlad och förvildad*”, ”*troligen förvildad*”, ” *förvildad eller ruderat*”, ”*troligen inplanterad och förvildad*”
introduced	”*införd*”, ”*adventiv*”, ”*adventiv, norrut tillfällig*”, ”*kulturspridd*”, ” *insådd med gräsfrö*”, ”*i park*”, ”*odlad*”, ”*planterad*”, ”*planterad eller förvildad*”, ”*införd, på avfallsplatser*”, ”*införd eller förvildad*”, ”*ursprungligen odlad*”, ”*införd på barlast*”, ” *troligen ursprungligen odlad*”, ” *inkommen i sen tid*”, ”*kulturflykting*”, ”*antropokor*”, ”*på avfallsplatser*”, ”*troligen ursprungligen inplanterad*”, ”*nyligen införd*”, ”*införd i sen tid*”, ”*kulturspridd*”, ”*som ogräs*”, ”*ej ursprunglig, utgången på de flesta lokalerna*”, ”*ofrivilligt planterad*”, ”*adventiv eller förvildad*”, ”*adventiv eller tillfällig*”, ”*införda former*”, ”*inkommen i sen tid*”, ”*troligen ursprungligen odlad*”, “*på barlast*”
transient	“*tillfällig*”, “*tillfällig eller utgången*”, “*tillfällig, kulturspridd*”, “*tillfälligt införd*”, ” *tillfällig på ruderatplatser*”, ”*avfallsplatser, tillfällig*”, ”*tillfälligt adventiv*”, ”*mer eller mindre tillfällig och numera sällan sedd*”, ”*tillfällig på avfallsplatser*”, ”*tillfällig i gräsmattor*”, ”*tillfällig, mestadels på barlast*”, ”*tillfällig, kulturspridd*”, ”*ruderat ofta tillfällig*”, “*tillfällig på barlast*”, ”*ruderat*”, ” *ruderat ofta tillfällig*”, ” *ruderat, mer eller mindre tillfällig*”
hybrid	”*hybrid*”
variety	The note in Swedish contains information on variety.
subspecies	The note in Swedish contains information on subspecies.
no information	Symbol with no information in the map legend.

Updated scientific nomenclature

To update the scientific nomenclature we matched the scientific names in the atlas with the Swedish taxonomical database Dyntaxa (SLU Artdatabanken 2022). Scientific synonyms with no match in Dyntaxa were checked using the Plants of the world online database (POWO 2022) for accepted scientific names. For scientific synonyms with no match in either Dyntaxa or POWO we checked the atlas (Hultén 1971) for listed scientific synonyms as well as synonyms listed in a later atlas published by Hultén (Hultén and Fries 1986) and checked these synonyms in POWO. The accepted names from POWO were then matched again in Dyntaxa. Some species which are not present in Sweden were not listed in Dyntaxa. For these species we have listed the scientific names accepted by the Global Biodiversity Information Facility (GBIF.org 2022). Detailed per species information is listed in the column *refDetailes* in the metadata file HultenMetadata.csv (Supporting Information: DataS1.zip). Some species, subspecies and varieties that were historically considered separate are now considered to belong to the same species. When this was the case, we merged the ranges into one polygon spatial feature file (GeoJSON). The species with merged ranges are listed in table 10. This information is also listed in *mergedRangesNote* in the metadata file HultenMetadata.csv (Supporting Information: DataS1.zip).

Table 10. Species where the range includes ranges of two or more species, subspecies or varieties that were historically considered separate, listed in the column *mergedRangesNote* in the metadata file HultenMetadata.csv (Supporting Information: DataS1.zip).

Scientific name	Names in Hultén (1971)
Alchemilla borealis	*Alchemilla borealis, Alchemilla transpolaris*
Betula pubescens	*Betula pubescens, Betula callosa*
Draba glabella	*Draba hirta, Draba hirta var. dovrensis*
Ficaria verna subsp. verna	*Ranunculus ficaria subsp. bulbifer, Ranunculus ficaria subsp. ficaria*
Helianthemum oelandicum	*Helianthemum oelandicum, Helianthemum italicum subsp. rupifragum*
Hieracium sect. Alpestria	*Hieracium - gruppen Dovrensia, Heriacium - gruppen Semidovrensia*
Juncus alpinoarticulatus subsp. rariflorus	*Juncus alpinus var. rariflorus, Juncus alpinus var. marshallii, Juncus alpinus*
Papaver dahlianum	*Papaver dahlianum, Papaver lujaurense*
Papaver lapponicum subsp. lapponicum	*Papaver chiinense, Papaver norvegicum, Papaver Tolmatchevii*
Papaver radicatum subsp. radicatum	*Papaver radicatum subsp. hyperboreum, Papaver radicatum subsp. macrostigma, Papaver radicatum subsp. ovatilobum, Papaver radicatum subsp. Subglobosum, Papaver Gjaerevollii*
Picea abies	*Picea abies, Picea abies var. arctica*

D. Computer programs and data-processing algorithms

HultenRangeMaps – R package

Methods employed to georeference the scanned maps, extract distribution data, clean data and convert data from raster to spatial feature (polygon) data are provided through the R package ‘HultenRangeMaps’ that is archived here (Supporting Information: DataS1.zip) and by the Swedish National Data Service at https://doi.org/10.58141/kvrm-hq58.

To aid spatial analyses, the package also provides a function to match the digitized distribution maps to any grid stored as a polygon spatial feature. The package provides examples on how to match the data to both the Swedish 10×10 km grid and the European 10×10 km grid. Given the scale of the original maps, the size of the dots representing isolated finds is approximately 16 km on the ground. This means that when we overlap the digitized and cleaned range maps (polygon features) with the 10×10 km Swedish National grid one ‘isolated find’ polygon will commonly overlap with 4-6 10×10 km grid cells, thus potentially over representing the historical range of a species. We overcome this by identifying ‘isolated finds’ polygons (polygons with circular shapes and of the right size) and reduce the area of these polygons by a 5000 m negative buffer.

This method can be employed to create historical presences of individual species and estimates of historical vascular plant biodiversity for any type of region within the spatial extent of the data (e.g. governmental regions, protected areas etc.) that are stored as a polygon spatial features. It is however important to note that, due to the effective resolution of 16×16 km of the atlas data as well as the gaps between the hatched lines marking continuous range areas (see Class II, part A – *Map resolution and legend*), using regions or grid-cells smaller than 10×10 km could result in regions or grids falling in between the lines. This would generate absences in areas where a species was considered present.

To ensure reproducibility, we have created a ‘mini’ repository with all the packages that the functions in ‘HultenRangeMaps’ require. The package README file includes examples of how to use the functions and how to install packages from local repositories. The package and associated ‘mini’ repository are archived on the Swedish National Data Service https://doi.org/10.58141/kvrm-hq58. Additional copies are available as follows.

HultenRangeMaps
https://github.com/ArnellM/HultenRangeMaps.git

HultenRangeMaps_miniCRAN_June2025
https://github.com/ArnellM/HultenRangeMaps_miniCRAN_June2025.git

Fiji ImageJ

The script used in the open-source biological-image analysis program Fiji ImageJ (Schindelin et al. 2012) to align the scanned maps is included in the Supporting Information (*align_bUnwarpJ.ijm*, DataS1.zip).

QGIS
The open-source geographic information system software QGIS (QGIS Development Team 2025) was used to manually georeference one reference map used in the automated georeferencing process as well as the manual data cleaning.

E. Archiving
Data are archived in the Supporting Information (DataS1.zip), as well as on the Swedish National Data Service (Arnell et al. 2026, https://doi.org/10.58141/y1x5-cx68). The R package 'HultenRangeMaps' and the associated 'mini' repository is archived in the Supporting Information (DataS1.zip) as well as on GitHub and on the Swedish National Data Service (Arnell 2026) https://doi.org/10.58141/kvrm-hq58. Contact Matilda Arnell (matilda.arnell@su.se) with any questions. Data and code are released under a Creative Commons Attribution 4.0 International (CC BY 4.0) license. When using this data set please cite both the dataset (Arnell et al. 2026), the original atlas (Hultén 1971) and this accompanying data paper.

Acknowledgements

The authors want to thank Christophe Avenel from the BioImage Informatics Facility at SciLifeLab for assistance with image registration of the scanned range maps. We also want to thank Irene Bisang for helping us try to retrieve the original maps. M.A and A.G.A. are supported by the Swedish research council Formas (2023-02457).

Literature Citations

Arganda-Carreras, I., C. O. S. Sorzano, R. Marabini, J. M. Carazo, C. Ortiz-de-Solorzano, and J. Kybic. 2006. Consistent and Elastic Registration of Histological Sections Using Vector-Spline Regularization. Pages 85–95 in R. R. Beichel and M. Sonka, editors. Computer Vision Approaches to Medical Image Analysis. Springer Berlin Heidelberg, Berlin, Heidelberg.

Arnell, H. W. 1923. Vegetationens årliga utvecklingsgång i Svealand. Meddelanden från Statens Meteorologisk-hydrografiska Anstalt 2:1, Stockholm.

Arnell, K. 1927. Vegetationens utvecklingsgång i Norrland. Meddelanden från Statens Meteorologisk-hydrografiska Anstalt 4:1, Stockholm.

Arnell, M. (2026). R package - HultenRangeMaps (Version 1). Stockholm University. https://doi.org/10.58141/kvrm-hq58

Arnell, K., and S. Arnell. 1930. Vegetationens utveckling i Götaland. Meddelanden från Statens Meteorologisk-hydrografiska Anstalt 6:2, Stockholm.

Arnell, M., A. G. Auffret, and K. Hylander. 2026. Historical distribution maps of vascular plants in Northern Europe (Version 1) [Data set]. Stockholm University. DOI: https://doi.org/10.58141/y1x5-cx68

Chamberlain, S., A. Teucher, and M. Mahoney. 2025. geojsonio: Convert Data from and to "GeoJSON" or "TopoJSON." R package version 0.11.3.9000, https://github.com/ropensci/geojsonio

GBIF.org. 2022. GBIF Home Page. https://www.gbif.org. Accessed 2022-11-25.

GDAL/OGR contributors. 2025. Geospatial Data Abstraction software Library. Open Source Geospatial Foundation. https://gdal.org

Hijmans, R. 2025a. raster: Geographic Data Analysis and Modeling. R package version 3.6-32. https://CRAN.R-project.org/package=raster

Hijmans, R. 2025b. terra: Spatial Data Analysis. R package version 1.8-60. https://CRAN.R-project.org/package=terra

Hjelt, H. 1888. Conspectus florae fennicae. Acta Societatis pro Fauna et Flora Fennica 5.

Hultén, E. 1950. Atlas of the distribution of vascular plants in northwestern Europe. Generalstabens Litografiska Anstalts Förlag, Stockholm.

Hultén, E. 1971. Atlas of the distribution of vascular plants in northwestern Europe. Second edition. Generalstabens Litografiska Anstalts Förlag, Stockholm.

Hultén, E., and M. Fries. 1986. Atlas of North European vascular plants : north of the Tropic of Cancer. Koeltz, Königstein.

Jessen, K. 1926. Oversigt over karplanternes udbredelse i Danmark. Botanisk tidsskrift 39.

Komarov, V. L. 1934. Flora SSSR. Izdatel'stvo Akademii nauk SSSR, Leningrad.

Krok, T. O. B. N. 1925. Bibliotheca botanica Suecana. Almqvist & Wiksell, Uppsala.

Moberg, A. 1860. Klimatologiska iakttagelser i Finland, föranstaltade och utgivna af Finska Vetenskaps-Societeten. 1. delen, År 1846-1855 / 1. Naturalhistoriska anteckningar. Finska vetenskaps-societeten, Helsingfors.

Moberg, A. 1885. Klimatologiska iakttagelser i Finland. 2:1, År 1856-1875 : Fenologiska anteckningar. Finska vetenskaps-societeten, Helsingfors.

O'Brien, J. 2023. gdalUtilities: Wrappers for "GDAL" Utilities Executables. R package version 1.2.5. https://CRAN.R-project.org/package=gdalUtilities

POWO. 2022. The International Plant Names Index and World Checklist of Vascular Plants. http://www.ipni.org and https://powo.science.kew.org/. Accessed 2022-11-25.

QGIS Development Team. 2025. QGIS Geographic Information System. Open Source Geospatial Foundation Project. http://qgis.osgeo.org

R Core Team. 2025. R: A language and environment for statistical computing. R Foundation for Statistical Computing, Vienna.

Rostrup, E., and C. A. Jörgensen. 1947. Den danske flora : en populær vejledning til at lære de danske planter at kende. 17th edition. Gyldendal, København.

Schindelin, J., I. Arganda-Carreras, E. Frise, V. Kaynig, M. Longair, T. Pietzsch, S. Preibisch, C. Rueden, S. Saalfeld, B. Schmid, J.-Y. Tinevez, D. J. White, V. Hartenstein, K. Eliceiri, P. Tomancak, and A. Cardona. 2012. Fiji: an open-source platform for biological-image analysis. Nature Methods 9:676–682.

SLU Artdatabanken. 2022. Dyntaxa - svensk taxonomisk databas. [Dataset]. https://artfakta.se/metadata/dyntaxa. Accessed 2022-09-09.

Sorzano, C. O. S., P. Thevenaz, and M. Unser. 2005. Elastic registration of biological images using vector-spline regularization. IEEE Transactions on Biomedical Engineering 52:652–663.

Steffen, H. 1940. Flora von Ostpreussen. Königsberg.
