# Supplementary material for: Historical distribution maps of vascular plants in Northern Europe
Source: Ecology. 2026 Sep 22;107(9):e70494. doi: 10.1002/ecy.70494 (PMC13597165; doi:10.1002/ecy.70494)
Supplement: Supplementary file 1 [file ECY-107-e70494-s001.zip › DataS1/HultenRangeMaps.zip!/HultenRangeMaps/docs/404.html]

Page not found (404) • HultenRangeMaps


 

Skip to contents


HultenRangeMaps
0.1


- Get started
- Reference

# Page not found (404)

Content not found. Please use links in the navbar.

Developed by Matilda Arnell.

Site built with pkgdown 2.1.3.
