# Supplementary material for: Historical distribution maps of vascular plants in Northern Europe
Source: Ecology. 2026 Sep 22;107(9):e70494. doi: 10.1002/ecy.70494 (PMC13597165; doi:10.1002/ecy.70494)
Supplement: Supplementary file 1 [file ECY-107-e70494-s001.zip › DataS1/HultenRangeMaps.zip!/HultenRangeMaps/docs/authors.html]

Authors and Citation • HultenRangeMaps    
Skip to contents


HultenRangeMaps
0.1


- Get started
- Reference

# Authors and Citation

## Authors

- **Matilda Arnell**. Author, maintainer.

## Citation

Arnell M (2025).
*HultenRangeMaps: Historical distribution maps of vascular plants in north western Europe*.
R package version 0.1.

```
@Manual{,
  title = {HultenRangeMaps: Historical distribution maps of vascular plants in north western Europe},
  author = {Matilda Arnell},
  year = {2025},
  note = {R package version 0.1},
}
```

## On this page

Developed by Matilda Arnell.

Site built with pkgdown 2.1.3.
