# Supplementary material for: Historical distribution maps of vascular plants in Northern Europe
Source: Ecology. 2026 Sep 22;107(9):e70494. doi: 10.1002/ecy.70494 (PMC13597165; doi:10.1002/ecy.70494)
Supplement: Supplementary file 1 [file ECY-107-e70494-s001.zip › DataS1/HultenRangeMaps.zip!/HultenRangeMaps/docs/index.html]

Historical distribution maps of vascular plants in north western Europe • HultenRangeMaps


 


Skip to contents


HultenRangeMaps
0.1


- Get started
- Reference

# HultenRangeMaps


This package contains the methods employed to digitise the historical range maps in the ‘Atlas of the distribution of vascular plants in northwestern Europe’ (Hultén 1971). Methods include georeferencing the scanned maps, extract range data, clean data and convert data from raster to spatial feature (polygon) data. A detailed description of how the original atlas was compiled as well as the method used to georeference and digitize the distribution maps can be found in Arnell et al. (xxxx). The dataset can be found here.   
 The package contains two functions to extract and clean distribution data from the scanned maps, one function to modify file names and one function to match the distribution data to polygon grids commonly used when analysing historical atlas data.

## Installation

This package can be installed from GitHub using the devtools package. To insure reproducability, we have created a ‘mini’ repository with all the packages that the functions in HultenRangeMaps require. The repository can be downloaded here. First, download the repository to your computer. Then, install the packages from your local repository using the code below.

```
# list of packages that the functions in HultenRangeMaps require
pkgs <- c("dplyr", "gdalUtilities", "geojsonio", "gtools", "igraph", "jpeg", "raster", "sf", "terra", "tidyr")

# path to where you have downloaded the local miniCran repository
pth <- "C:/miniCran/HultenRangeMaps_miniCRAN_June2025"

# install packages from your local miniCRAN repository
install.packages(pkgs, 
                 repos = paste0("file:///", pth),
                 type = "source")

# install HultenRange maps
library(devtools)
install_github("ArnellM/HultenRangeMaps")
library(HultenRangeMaps)
```

## Examples

Examples of each function can be found in the package vignette.

## References

Arnell et al. (XXXa)

Arnell et al. (XXXb)

Hultén, E. (1971). Atlas of the distribution of vascular plants in northwestern Europe (2nd ed.). Generalstabens Litografiska Anstalts Förlag.

## License

- `use\_mit\_license()`, `use\_gpl3\_license()` or friends to pick a license

## Citation

- Citing HultenRangeMaps

## Developers

- Matilda Arnell   
   Author, maintainer

Developed by Matilda Arnell.

Site built with pkgdown 2.1.3.
