# Supplementary material for: Historical distribution maps of vascular plants in Northern Europe
Source: Ecology. 2026 Sep 22;107(9):e70494. doi: 10.1002/ecy.70494 (PMC13597165; doi:10.1002/ecy.70494)
Supplement: Supplementary file 1 [file ECY-107-e70494-s001.zip › DataS1/HultenRangeMaps.zip!/HultenRangeMaps/docs/reference/fixFileNames.html]

Cange filenames to match Dyntaxa — fixFileNames • HultenRangeMaps    
Skip to contents


HultenRangeMaps
0.1


- Get started
- Reference

# Cange filenames to match Dyntaxa

`fixFileNames.Rd`

In the Swedish Taxonomic Database Dyntaxa, hybrid species are denoted by the symbol ×. To facilitate file handling the symbol × has been replaced by the letter x in the database file names. This function changes the letter x in the file names back the symbol × so that the file names are identical to the Dyntaxa scientific names.

## Usage

```
fixFileNames(path, pattern = "*.geojson")
```

## Arguments

path
:   file path folder with range map database

pattern
:   file extension. Default is patterm = "\*.geojson"

## Value

new filenames

## On this page

Developed by Matilda Arnell.

Site built with pkgdown 2.1.3.
