# Supplementary material for: Historical distribution maps of vascular plants in Northern Europe
Source: Ecology. 2026 Sep 22;107(9):e70494. doi: 10.1002/ecy.70494 (PMC13597165; doi:10.1002/ecy.70494)
Supplement: Supplementary file 1 [file ECY-107-e70494-s001.zip › DataS1/HultenRangeMaps.zip!/HultenRangeMaps/docs/reference/index.html]

Package index • HultenRangeMaps    
Skip to contents


HultenRangeMaps
0.1


- Get started
- Reference

# Package index

## All functions

`fixFileNames()`
:   Cange filenames to match Dyntaxa

`mapCleanConvert()`
:   Clean raster and convert to polygon feature

`mapDig()`
:   Digitise scanned range maps

`rangeToGrid()`
:   Match species' ranges to (national) grid

Developed by Matilda Arnell.

Site built with pkgdown 2.1.3.
