# Supplementary material for: Historical distribution maps of vascular plants in Northern Europe
Source: Ecology. 2026 Sep 22;107(9):e70494. doi: 10.1002/ecy.70494 (PMC13597165; doi:10.1002/ecy.70494)
Supplement: Supplementary file 1 [file ECY-107-e70494-s001.zip › DataS1/HultenRangeMaps.zip!/HultenRangeMaps/docs/reference/mapCleanConvert.html]

Clean raster and convert to polygon feature — mapCleanConvert • HultenRangeMaps    
Skip to contents


HultenRangeMaps
0.1


- Get started
- Reference

# Clean raster and convert to polygon feature

`mapCleanConvert.Rd`

Cleans the digitised range map by removing (most of the) map border, removing small inconsistencies by 'clumping' and 'sifting' and converts the raster map to a polygon feature.

## Usage

```
mapCleanConvert(x)
```

## Arguments

x
:   digitised map (RasterLayer)

## Value

a polygon feature (SpatVector)

## On this page

Developed by Matilda Arnell.

Site built with pkgdown 2.1.3.
