# Supplementary material for: Historical distribution maps of vascular plants in Northern Europe
Source: Ecology. 2026 Sep 22;107(9):e70494. doi: 10.1002/ecy.70494 (PMC13597165; doi:10.1002/ecy.70494)
Supplement: Supplementary file 1 [file ECY-107-e70494-s001.zip › DataS1/HultenRangeMaps.zip!/HultenRangeMaps/docs/reference/mapDig.html]

Digitise scanned range maps — mapDig • HultenRangeMaps    
Skip to contents


HultenRangeMaps
0.1


- Get started
- Reference

# Digitise scanned range maps

`mapDig.Rd`

Takes a scanned range map (raster stack) with three bands (Red-Green-Blue) and extracts the range information by taking the green raster band and setting all pixel values up to 150 to 1 and all other to 0. The pixel range 0-150 in green raster band represents the dark red areas in the scanned map.

## Usage

```
mapDig(x, value = 150)
```

## Arguments

x
:   scanned range map (RGB rasterStack).

value
:   maximum pixel value that delimits the pixel values representing the range information in the scanned maps. Default value is x=150.

## Value

A raster layer.

## On this page

Developed by Matilda Arnell.

Site built with pkgdown 2.1.3.
