# Supplementary material for: Historical distribution maps of vascular plants in Northern Europe
Source: Ecology. 2026 Sep 22;107(9):e70494. doi: 10.1002/ecy.70494 (PMC13597165; doi:10.1002/ecy.70494)
Supplement: Supplementary file 1 [file ECY-107-e70494-s001.zip › DataS1/HultenRangeMaps.zip!/HultenRangeMaps/docs/reference/rangeToGrid.html]

Match species' ranges to (national) grid — rangeToGrid • HultenRangeMaps    
Skip to contents


HultenRangeMaps
0.1


- Get started
- Reference

# Match species' ranges to (national) grid

`rangeToGrid.Rd`

Match species' ranges to (national) grid

## Usage

```
rangeToGrid(path, pattern = "*.geojson", grid = SweNationalGrid)
```

## Arguments

path
:   file path to folder with range map database

pattern
:   file extension. Default is patterm = "\*.geojson"

grid
:   (national) grid polygon feature

## Value

a list of data frames with per species per grid cell historical presence

## On this page

Developed by Matilda Arnell.

Site built with pkgdown 2.1.3.
